# Two years experience with quality assurance protocol for patient related Rapid Arc treatment plan verification using a two dimensional ionization chamber array

**DOI:** 10.1186/1748-717X-6-21

**Published:** 2011-02-22

**Authors:** Daniela Wagner, Hilke Vorwerk

**Affiliations:** 1 Department of Radiotherapy and Radiooncology, University Hospital Goettingen, Robert-Koch-Str. 40, 37075 Goettingen, Germany

## Abstract

**Purpose:**

To verify the dose distribution and number of monitor units (MU) for dynamic treatment techniques like volumetric modulated single arc radiation therapy - Rapid Arc - each patient treatment plan has to be verified prior to the first treatment. The purpose of this study was to develop a patient related treatment plan verification protocol using a two dimensional ionization chamber array (MatriXX, IBA, Schwarzenbruck, Germany).

**Method:**

Measurements were done to determine the dependence between response of 2D ionization chamber array, beam direction, and field size. Also the reproducibility of the measurements was checked. For the patient related verifications the original patient Rapid Arc treatment plan was projected on CT dataset of the MatriXX and the dose distribution was calculated. After irradiation of the Rapid Arc verification plans measured and calculated 2D dose distributions were compared using the gamma evaluation method implemented in the measuring software OmniPro (version 1.5, IBA, Schwarzenbruck, Germany).

**Results:**

The dependence between response of 2D ionization chamber array, field size and beam direction has shown a passing rate of 99% for field sizes between 7 cm × 7 cm and 24 cm × 24 cm for measurements of single arc. For smaller and larger field sizes than 7 cm × 7 cm and 24 cm × 24 cm the passing rate was less than 99%. The reproducibility was within a passing rate of 99% and 100%. The accuracy of the whole process including the uncertainty of the measuring system, treatment planning system, linear accelerator and isocentric laser system in the treatment room was acceptable for treatment plan verification using gamma criteria of 3% and 3 mm, 2D global gamma index.

**Conclusion:**

It was possible to verify the 2D dose distribution and MU of Rapid Arc treatment plans using the MatriXX. The use of the MatriXX for Rapid Arc treatment plan verification in clinical routine is reasonable. The passing rate should be 99% than the verification protocol is able to detect clinically significant errors.

## Introduction

Rapid Arc radiotherapy technology from Varian Medical Systems is one of the most complex delivery systems currently available, and achieves an entire intensity-modulated radiation therapy (IMRT) treatment in a single gantry rotation around the patient. Three dynamic parameters can be continuously varied to create IMRT dose distributions: speed of rotation, beam shaping aperture and delivery dose rate [1]. The variation of three dynamic parameters is used to cover the planning target volume with clinical acceptable dose and to spare the organs at risk (OAR) and normal tissue. Due to the volumetric single arc the treatment can be performed in less time than IMRT treatment. Some studies compared the dose to OAR, healthy tissue sparing, and target coverage of Rapid Arc to conventional forwardly planed radiotherapy technique, fixed field IMRT, Helical Tomotherapy, and Intensity Modulated Proton therapy [2-17].

Presupposition for clinically significant advances in the management of cancer is the correct calculation of the dose distribution and the correct treatment delivery. Gagne at al. have shown that the calculation of the dose distribution can be performed with a clinical acceptable accuracy using the algorithm AAA (anisotropic analytical algorithm [18,19]) with a resolution of 2.5 mm or better [20]. Ling et al. have shown that the DMLC movement, variable dose-rates and gantry speeds can be precisely controlled during Rapid Arc [21].

In opposite to 3D conventional treatment techniques in dynamic treatment techniques the verification of the MU is much more complex. Therefore the dose distribution is verified using 2D or 3D measuring devices like 2D ionization chamber arrays or phantoms equipped with radiographic films. In the past some investigations were done to ascertain the potential of different types of 2D ionization chambers for IMRT verification measurements [22-36].

The purpose of this study was to analyze the potential of the MatriXX for patient related verification of Rapid Arc treatment plans. Therefore some preparing measurements were done. We determined the dependence between response of the MatriXX, beam direction, and field size. Also we repeated 2D dose distribution measurements ten times and compared each measurement with the first one to check the reproducibility of the method.

## Materials and methods

### Two dimensional ionization chamber array

The two dimensional ionization chamber array consists of a 32 × 32 matrix of 1024 parallel plate ionization chambers. The ionization chambers are arranged in a square of 24 cm × 24 cm as active measuring area. Each chamber has 0.4 cm diameter and 0.55 cm height. The distance between each ionization chamber is 0.75 cm from centre to centre of adjacent chambers. The sensitive volume of each single ionization chamber is 0.07 cm^3^. Each of the 1024 independent ionization chambers is read out with a custom microelectronics chip.

### Preparing measurements

To analyse the potential of the MatriXX for verification of Rapid Arc treatment plans two measurement series were accomplished. First the dependence of MatriXX response of beam direction and field size were analysed. Therefore the MatriXX was irradiated with unblocked photon arc fields with field sizes of 3 cm × 3 cm, 5 cm × 5 cm, 10 cm × 10 cm, 20 cm × 20 cm, 24 cm × 24 cm, and 30 cm × 30 cm. The unblocked photon arc fields were irradiated using a full rotation of the gantry around the MatriXX (start angle 181°, stop angle 179°, counter clockwise, Varian scale IEC 601). Second the reproducibility was checked for field size of 10 cm × 10 cm. Therefore the measurement was compared with repeated measurements with the same setup using the gamma evaluation method with the criteria 3% und 2 mm, no threshold, 2D global gamma index [37]. The measurement was repeated ten times. The measurements took place at the Clinac 2300 C/D (Varian Medical System, Palo Alto, CA, USA). For all measurements a photon energy of 6 MV_photons _was used. 500 MU were applied for all measurements. The MatriXX was used in the acquisition mode "Movie Mode". The sampling time was set to 200 ms, the maximum number of sample to 5, and the number of movie images to 2000. The measured matrix was interpolated linear to 1 mm and was scaled relative to maximum. All measurements were normalized to maximum dose. For the treatment the verification plan has to be prepared within the record and verify system. The manufacturer declared a warm up time of 15 min and pre-irradiation with 10 Gy before measurement.

### Patient treatment plans

Different patient treatment plans were used, 53 treatment plans of the head region, 68 treatment plans of the head and neck region, and 312 treatment plans of the pelvis region. A total of 433 different treatment plans in complexity with 598 arcs were measured and analyzed. For treatment of gliomas a total dose (TD) of 60 Gy with a single dose (SD) of 2.0 Gy was used. The other treatments in the head regions were applied with a TD of 30 Gy (SD 2.75 Gy) to the whole brain with a concomitant boost with a TD of 45 Gy (SD 3.75 Gy). Additional the boost plans of cerebral metastases with a TD of 9 Gy or 15 Gy (SD 2.5 Gy or 3.0 Gy, respectively) are analysed. Head and neck cancer patients were treated using an integrated protocol with a TD 54 Gy (SD 1.8 Gy) to lymph node regions, which were possible involved, and a TD 57.6 Gy (SD 1.92 Gy) to lymph node regions, which are involved with a high possibility. The region of the primary tumour was treated with a TD of 66 Gy (SD 2.2 Gy) for treatment with a curative intent and with a TD of 62.4 Gy (SD 2.08 Gy) for adjuvant intent. In the pelvis region patient with rectal cancer (neo adjuvant treatment with TD 50.4 Gy, SD 1.8 Gy), cervical cancer (adjuvant treatment with TD 50.4 Gy, SD 1.8 Gy) and prostate cancer were analysed. The TD for patient with prostate cancer differed between 60 Gy to 72 Gy (SD 2.0 Gy). Some patient received additional and concomitant treatment of the lymph node region with a TD of 45 Gy (SD 1.8 Gy). For all treatment plans photon energy of 6 MV_Photons _with the dose rate of 600 MU/min (800 MU/min for field size smaller than 15 cm × 15 cm and energy mode 6 MV_photon_s SRS) were used. The treatment plans were optimized for single or double arc delivery (s. table 1 for average field size and for average MU). For single arc delivery the gantry rotated clockwise around the patient, for double arc delivery first clockwise and second counter clockwise. The start angle for single arc in clockwise direction ranged between 181° and 270°, and the stop angle between 90° and 179°. The start angle for the second arc in counter clockwise direction ranged between 90° and 179°, and the stop angle for the second arc in counter clockwise direction between 181° and 270°. For each treatment plan 177 control points were set. The dose distribution for all plans were calculated with Eclipse treatment planning system (TPS) from Varian Medical Systems, version 8.5; using AAA algorithm with a grid size of 0.2 cm × 0.2 cm × 0.2 cm. The AAA is a 3D pencil Beam convolution/superposition algorithm that uses separate Monte Carlo derived modelling for primary photons, scattered extra-focal photons, and electrons scattered from the beam limiting devices [18,19]. The treatment couch structures (exact couch, Varian Medical Systems, Palo Alto, CA, USA) were considered during the calculation process.

**Table 1:** Results.

region	number of monitor units	Treatment time [sec]	passing rate	PTV [ml]	field size cm
head	341 ± 221	0.99 ± 0.12	99.88 ± 0.19	388.2 ± 539.6	14.7 ± 5.5
head and neck	191 ± 102	0.85 ± 0.01	99.80 ± 0.39	477.8 ± 429.8	20.1 ± 4.8
prostate	300 ± 123	1.00 ± 0.06	99.54 ± 0.21	717.2 ± 617.5	15.6 ± 4.9
abdomen	231 ± 28	0.92 ± 0.05	99.76 ± 0.32	1032.6 ± 500.8	20.7 ± 2.3

range					

head	118 - 1717	0.78 - 1.09	99.11 - 100.00	7.9 - 1895.0	7.0 - 24.0
head and neck	118 - 693	0.84 - 0.85	97.04 - 100.00	22.3 - 1916.3	7.0 - 24.0
prostate	129 - 906	0.91 - 1.08	98.84 - 100.00	37.6 - 3559.8	7.2 - 24.0
abdomen	195 - 274	0.89 - 0.96	99.15 - 100.00	894.5 - 2140.6	19.0 - 24.0

The columns show (left to right) the region of the localization of the PTV, the mean MU used for the treatment and its standard deviation, the mean treatment time per arc and its standard deviation, the mean passing rate and its standard deviation, the mean volume of the PTV and its standard deviation, and the mean field size by jaws and its standard deviation. The first three rows give the mean results for the different regions. The last three rows show the range of each region.

### Verification treatment plans

The patient Rapid Arc treatment plan was projected on the CT scan of the MatriXX including 4 cm polymethylmethacrylate (PMMA) above and underneath the active measuring area to account for build up and backscatter. The isocenter was positioned at the centre of the active measuring area. For calculation of the dose distribution the TPS Eclipse using the AAA algorithm, version 8.5 with a grid size of 0.2 cm × 0.2 cm × 0.2 cm was used. The treatment couch structures were considered during the calculation process.

### Analysis

The 2D dose distribution in the active measuring area in the frontal CT slice of the verification plan was exported with the resolution of 1 mm and imported into the software OmniPro. The measured dose distribution was generated during single or double rotation of the gantry around the MatriXX. The acquisition mode, scaling mode, sampling time, number of samples, number of movie images and interpolation algorithm was set as described above. The analysis was made using gamma evaluation method [37] to compare measured and calculated dose distribution. The gamma evaluation criteria were 3% and 3 mm, no threshold, 2D global gamma index. For the analysis of the gamma evaluation result the histogram of the gamma evaluation was displayed. The histogram of the gamma evaluation plotted the number of pixel against the gamma value. The total number of pixel with a gamma value above 1 was divided by the total number of pixel within the region of interest (ROI). The ROI was set to field size +1 cm.

## Results

### Preparing measurements

The MatriXX response agrees within 99% of pixel with gamma evaluation value beneath 1 for field sizes between 7 cm × 7 cm and 24 cm × 24 cm for measurements of single arc. For smaller and larger field sizes then 7 cm × 7 cm and 24 cm × 24 cm the response was less than 99% of the pixel with gamma evaluation value beneath 1. The passing rate was 93.8% for field size 3 cm × 3 cm, 98.3% for field size 5 cm × 5 cm, and 82.2% for field size 30 cm × 30 cm, respectively. The reproducibility was within a passing rate of 99% and 100% (range 99.4% and 100.0%).

### Verification treatment plans

The mean treatment time was 1.05 min for all patients, ranging from 0.78 min to 1.56 min. We do not use full rotation of the gantry around the patient for all treatments. If possible we spared the treatment couch and OAR, which have low dose tolerance like lenses. The system tried to move the gantry with maximum speed if the leafs of the multi leaf collimator (MLC) could move into the given position that fast and the MU could be delivered that fast. If the dose rate reached the maximum of 600 MU/min (800 MU/min for field sizes smaller than 15 cm × 15 cm and energy mode 6 MV_photons _SRS) the gantry speed was reduced.

After measurement, both - measured and calculated dose distribution - were compared using gamma evaluation method implemented in the software OmniPro. Using the histogram the distribution of the gamma evaluation was displayed. The results of the ratio of the number of pixel beneath the gamma evaluation value of 1 divided by the total number of pixel within the ROI is shown in table 1.

The passing rate was between 99.0% and 100.0% in 431 of 433 cases. In two cases - one head and neck and one prostate case - the passing rate was 97.7% and 98.8%, respectively. In 53 head cases the mean passing rate was 99.88% ± 0.19% with PTV volume sizes ranging between 7.9 ml and 1895.0 ml and the resulting square field sizes between 7.0 cm and 24.0 cm. The mean passing rate in 68 head and neck cases was 99.80% ± 0.39% with PTV volume size ranging between 22.3 ml and 1916.3 ml and the resulting square field sizes between 7.0 cm and 24.0 cm. For 312 cases in pelvis region the mean passing rate was 99.54% ± 0.21% with PTV volume sizes ranging between 37.6 ml and 3559.8 ml and the resulting square field sizes between 7.2 cm and 24.0 cm.

**Figure 1:**
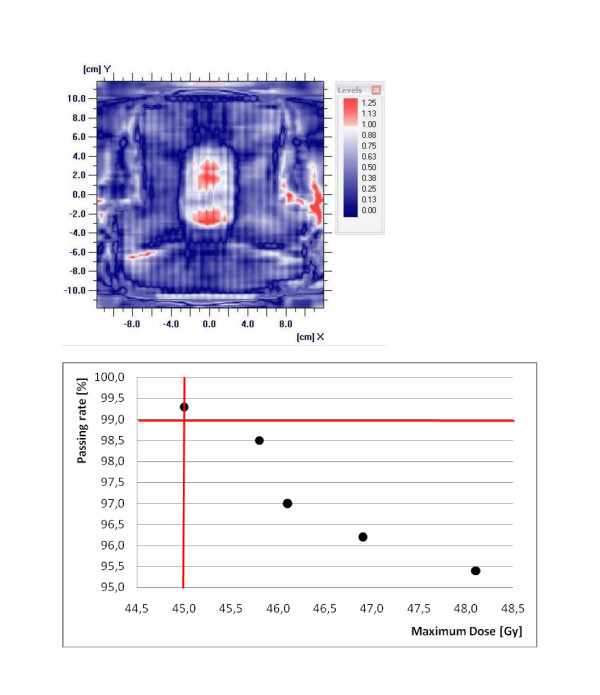
**a) Example of measured 2D dose distribution of a head and neck case**. The MLC positions were changed in the region of the spinal cord to got higher dose to the spinal cord which could not be clinically tolerated. b) Passing rate against the maximum dose to the spinal cord of the same head and neck case. 4 Rapid Arc treatment plans were generated by changing the MLC positions. The changed Rapid Arc treatment plans were compared to the original Rapid Arc treatment plan. The original Rapid Arc treatment plan showed a passing rate of 99.3%. The lines indicate the in our clinic tolerated limits: 45 Gy maximum dose to the spinal cord and 99% passing rate.

To check if the verification protocol is able to detect clinically significant errors the original patient Rapid Arc treatment plan was manipulated. Therefore the MLC position (Millennium 120 Multi leaf collimator, Varian Medical Systems, Palo Alto, CA, USA) was changed and the dose distribution was calculated with the changed MLC position. To change the MLC position single leafs has to be set to another position at all 177 control points using the MLC movement tool of the TPS. The 2D dose distribution was measured and compared with the original, unchanged 2D dose distribution. If the MLC position was changed in a way that the dose distribution was changed clinically significant and therefore the probability of toxicity was increased the passing rate was less than 99% with the settings of the MatriXX mentioned above (s. figure 1). Clinically significant means increasing the dose to the OAR higher than the given limits by Emami et al. [38], and decreasing the dose to the PTV according to ICRU-50 report.

### Uncertainty budget

Since the comparison of measured and calculated 2D dose distribution was considered as the end result, the following sources contributing to the overall uncertainty of the result were identified:

• MatriXX measurement method

• Monitor output fluctuation of treatment machine

• Dose calculation of the treatment planning system

• Specifications of treatment machine

In our study, the uncertainty components for MatriXX measurement method had to be taken into account due to positioning of MatriXX using the isocentric laser system in treatment room, the broadening of the penumbra due to the volume effect of the ionization chambers which act as low pass filter, and an additional component for response of ionization chamber reading. In addition, the daily monitor output fluctuation of the treatment machine varies up to 0.75% (daily measurements with ionization chamber). The manufacturer of the treatment machine specifies dose stability during gantry rotation to 2%; accuracy of gantry, collimator, and couch rotation to 0.75 mm; and accuracy of MLC positioning to 1 mm. For the accuracy of the dose calculation, the manufacturer specifies 1.0% for unblocked photon fields. The uncertainty of the treatment machine's basic data measurements had to be taken into consideration within 2 mm. The consideration of 2 mm contained the exact positioning of the ionization chamber during basic data measurements for the TPS before clinical operation. The different contributions are listed in table 2.

**Table 2:** Uncertainty components for the verification method.

Uncertainty budget	
Components for measurement	Uncertainty
	
MatriXX Ionization chambers	2%
broadening of penumbra (low pass filter)	1 mm
positioning of IMRT-MatriXX	1 mm
monitur output fluctuation	0.75%
dose stability during gantry rotation	2%
stability gantry, collimator, and couch rotation	0.75 mm
MLC positioning	1 mm
	

**Total**	**2.9%, 1.9 mm**
	

Components for calculation	Uncertainty

	
dose calculation of treatment planning system	1%
Basic data measurements	2 mm
	

**Total**	**1%, 2 mm**

The common used gamma evaluation criteria 3% and 3 mm were assumed to be sufficient for the evaluation of the measured and calculated 2D dose distribution in the active measurement area of the MatriXX.

## Discussion

We investigated this study to generate a patient related verification procedure for Rapid Arc treatment plans.

Before we started to generate the verification protocol we investigated measurements to analyse the potential of the MatriXX for unblocked photon arc fields. The MatriXX response showed good agreements between calculated and measured dose distribution for field sizes of 7 cm × 7 cm and 24 cm × 24 cm with a passing rate between 99% and 100%. Higher aberrations were found for smaller field sizes than 7 cm × 7 cm and for larger field sizes than 24 cm × 24 cm. During Rapid Arc verification measurement we measured the whole dose distribution which consists of 177 control points (177 beam directions with different MLC shapes and gantry speeds between each beam direction). 2/177 beams irradiated perpendicular through the MatriXX. In addition a range of control points irradiated near lateral through the MatriXX. The advantage of the MatriXX is cylindrical parallel plate chambers. Our results showed good agreement between measured and calculated dose distribution in the active measurement area. We assume that the TPS considered the beam angle dependence of the MatriXX correctly. Correctly in this context means that the angular dependence is clinically tolerable for field sizes between 7 cm × 7 cm and 24 cm × 24 cm.

In our study we projected the patient Rapid Arc treatment plan with its MLC shape, gantry speed and dose rate parameters on the CT dataset of the MatriXX including 4 cm build up and 4 cm backscatter material as well as the treatment couch structures. The electron density of the different parts of the MatriXX as well as the treatment couch was considered during dose distribution calculation. Using the TPS Eclipse V. 8.5 and above it is possible to insert Varian treatment couch types to the CT dataset. The absorption of the treatment couch is considered by giving the system Hounsfield Units (HU) for each part of the treatment couch like couch top and rails. The correct HU were determined by comparison of measured values using an ionization chamber and calculated values by TPS [for more details s. [39]]. In the past several studies were published which showed that the treatment couch attenuation is up to 3% for beam direction 180° and up to 9% for oblique beam directions [40-45] and needs to be considered.

According to the study of Ling et al. [21] quality assurance of treatment machine especially for Rapid Arc was done monthly as well as weekly measurements of absolute dose of arc fields and dynamic MLC fields using ionization chamber. Due to our quality assurance we could be sure that the treatment machine delivered complex Rapid Arc plans correctly if the 2D dose distribution was within the passing rate of 99% using the presented method.

Wolfsberger et al. presented recently their method for IMRT and Rapid Arc quality assurance [36]. They showed in their study that the MatriXX response is dependent on beam directions. The dependency was 7% to 11% for perpendicular and oblique beam directions and need to be corrected. They suggest correcting the angular dependence using correction factors for each beam angle. Popple et. al. published their first experience with patient related quality assurance of dynamic treatment techniques (IMRT and Rapid Arc) of 52 cases [46]. In their study they considered the angular dependence of the 2D ionization chamber array as well. In opposite to Wolfsberger et. al. they corrected the angular dependence using a special formed phantom (Multicube, IBA, Schwarzenbruck) which considered for the angular dependence. Van Esch et. al. considered the angular dependence of the 2D ionization chamber array in the same way using a special formed phantom [27]. Our presented method allows the quality assurance of Rapid Arc treatment plans prior to treatment. The method was tested for quality assurance of 433 treatment plans with different complexity. We projected the patient treatment plan on the CT dataset of the MatriXX including the treatment couch structures and calculated the dose distribution using the AAA algorithm. Due to our results we conclude that the angular dependence may be considered correctly/clinically tolerable in the TPS if the CT dataset of the measurement device including 4 cm build up and 4 cm backscatter material, the treatment couch structures, and the AAA algorithm with a grid size of 0.2 cm × 0.2 cm × 0.2 cm are used for field sizes between 7 cm × 7 cm and 24 cm × 24 cm.

In different studies [for example [27,36], and [46]] the angular dependence of 2D ionization chamber array are determined and considered in different ways. All studies showed that with their presented method the angular dependence was considered correctly to use the measurement device for the quality assurance of dynamic treatment techniques. In our study we considered for the angular dependence by calculation of the dose distribution on the CT dataset of the MatriXX, by considering the treatment couch structures during the calculation process, by using all beam directions of all 177 control points, by using 4 cm PMMA for build up and 4 cm PMMA for backscatter, and by using the AAA algorithm with a grid size of 0.2 cm × 0.2 cm × 0.2 cm. Due to this setup the effect of angular dependence of the MatriXX is clinically tolerable for 2D dose distribution comparison using the gamma evaluation method with criteria 3% and 3 mm.

To characterize the angular sensitivity of the MatriXX we have done measurements before starting to implement a verification protocol for patient related Rapid Arc treatment plan verifications. We have used a different way to characterize the angular dependence and field size dependence of the MatriXX. We combined both end results in one test to get one combined end result. We felt that this method is adequate because during the Rapid Arc treatment and the measurement of the 2D dose distribution using the MatriXX the dependence of angular beams and dependence of field sizes are combined as well. Different studies have shown dependence of beam angle and treatment couch considering as single end result, but did not combine different end results. By combining different end results in one test setup the potential of the measurement device can be seen easily for the setup which will be used during quality assurance measurements.

Herzen et al. analysed the dose and energy dependence of the MatriXX. They showed that the detector's response was linear with dose and energy independent [30]. The purpose of the development of two dimensional detector arrays was to ease the two dimensional verifications of fields with complex shapes and large gradients [26]. Since two dimensional detector arrays have been developed, these systems have been used for quality control and verification of IMRT. The results of some studies were in good agreement with calculations performed with the TPS and with the standard dosimetric tools, i.e., films or various point dose detectors [22-24].

The MatriXX has the potential for Rapid Arc treatment plan verifications. If the MLC position was changed in a way that the dose distribution was changed clinically significant, the passing rate was less than 99% with gamma criteria 3% and 3 mm for the presented method (s. figure 1). For a passing rate below 99% the optimization and calculation of the patient Rapid Arc treatment plan has to be redone using other constraints for the optimization process to smooth the dose gradients.

The measured and calculated doses were normalized relative to dose maximum. By the normalization process the deviation in the gamma evaluation method were suppressed in low dose regions. It has to be taken into account that OAR are presented in the low dose region and the passing of the gamma evaluation criteria 3% and 3 mm may result in larger deviation in this regions. However in the most cases the gradients and modulations were not really high using the treatment protocols for the presented cases as described above and Rapid Arc treatments. Therefore the normalization method could be tolerated.

We set the passing rate to 99% than the verification protocol is able to detect clinically significant errors as the manipulated measurements showed. Some of the irradiated ionization chambers of the MatriXX may have significant larger deviations. This larger deviation may lead to patient mistreatment. It has to be taken into account that the mistreatment was true for the actual fraction. However the patient was setup several times for the treatment and the single larger deviations will be spread.

For IMRT treatment plans the manufacturer has implemented the use of the portal imager as measuring device. It is quite easy to verify IMRT treatment plans with portal dosimetry because of no need to set up an external measuring device. Therefore the setup error has not to be taken into account using the laser system in the treatment room. The manufacturer is questioned to provide the portal dosimetry system for Rapid Arc verifications as well.

## Conclusion

The method is easy to implement into clinical routine. Verifying 2D dose distribution and MU with the MatriXX for Rapid Arc treatment plans is reasonable. The passing rate should to be 99% to detect clinically significant errors using the gamma criteria 3% and 3 mm, 2D global gamma index.

## Competing interests

The authors declare that they have no competing interests.

## Authors' contributions

All authors contributed substantially to the manuscript: DW contributed in the conception and realisation of the study and data acquisition and analysis, and HV with the drafting and revising of the article. Both authors read and approved the final manuscript.
